# Indoor and Outdoor Backpack Mapping with Calibrated Pair of Velodyne LiDARs

**DOI:** 10.3390/s19183944

**Published:** 2019-09-12

**Authors:** Martin Velas, Michal Spanel, Tomas Sleziak, Jiri Habrovec, Adam Herout

**Affiliations:** 1 Department of Computer Graphics and Multimedia, Faculty of Information Technology, Brno University of Technology, Bozetechova 1/2, 612 66 Brno, Czech Republic; 2 Geodrom Company, Bohunicka 81, 619 00 Brno, Czech Republic

**Keywords:** backpack laser mapping, BIM, Velodyne LiDAR, point cloud, GNSS, IMU, sensor calibration, surveying

## Abstract

This paper presents a human-carried mapping backpack based on a pair of Velodyne LiDAR scanners. Our system is a universal solution for both large scale outdoor and smaller indoor environments. It benefits from a combination of two LiDAR scanners, which makes the odometry estimation more precise. The scanners are mounted under different angles, thus a larger space around the backpack is scanned. By fusion with GNSS/INS sub-system, the mapping of featureless environments and the georeferencing of resulting point cloud is possible. By deploying SoA methods for registration and the loop closure optimization, it provides sufficient precision for many applications in BIM (Building Information Modeling), inventory check, construction planning, etc. In our indoor experiments, we evaluated our proposed backpack against ZEB-1 solution, using FARO terrestrial scanner as the reference, yielding similar results in terms of precision, while our system provides higher data density, laser intensity readings, and scalability for large environments.

## 1. Introduction

**Figure 1:**
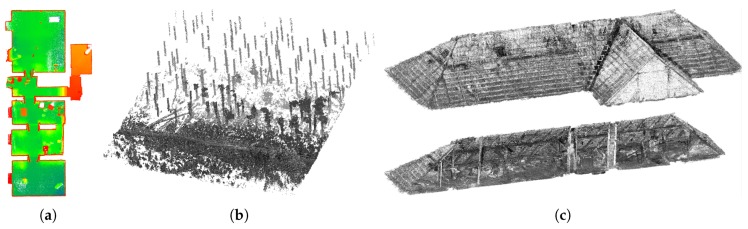
The motivation and the results of our work. The reconstruction of indoor environments (**a**) is beneficial for inspection, inventory checking and automatic floor plans generation. 3D maps of forest environments (**b**) is useful for quick and precise estimation of the biomass (timber) amount. The other example of 3D LiDAR mapping deployment is preserving cultural heritages or providing models of historical building, e.g., the roof in (**c**).

In recent years, the LiDAR (Light Detection And Ranging) technology has become very popular in the field of geodesy and related fields, where the availability of 3D models of outdoor or indoor environments can be beneficial: e.g., forestry, architecture, preserving cultural heritage, construction monitoring, etc. The examples of reconstructions from similar practical applications can be found in Figure 1. Using 3D mapping can also be beneficial for time and cost reduction. The same model can be shared among different professionals in different fields of expertise without the need for personal inspection and measuring at a given place individually.

This demand causes a huge interest in developing solutions that would be able to capture the reality and provide reliable 3D reconstructions out of the box. However, there are also other requirements for such a system.

The data acquisition process has to be quick and the planning of fieldwork should be minimized. This requirement discriminates solutions based on static terrestrial lasers (e.g., Leica and Riegl of FARO companies), requiring detailed planning of the data acquisition and manual system set up on a tripod within multiple convenient viewpoints across the scene.

The solution has to be mobile and easy to handle. This naturally leads to the preference of human carried (backpack or handheld) solutions instead of terrestrial or vehicle based solutions, such as NavVis [1], which, for example, does not support traversing tilted surfaces such as ramps.

However, the necessity for reliability in terms of resulting model precision is in contradiction with these two requirements. Stationary terrestrial LiDAR solutions require time demanding scanning process while providing a great accuracy (in order of millimeters) because of fewer degrees of freedom. Although, for many applications listed above, there is no need for such precision, our goal is the difference between the reality and the resulting 3D model below 5 cm. This value was requested by the experts in the field of geodesy with whom we consulted.

In the practical applications, completeness of the final map should also be guaranteed because it might be difficult to repeat the scanning. The operator has to be aware of the fact that all necessary data of the whole environment were acquired. We fulfilled this requirement by providing a live preview of the collected data.

**Figure 2:**
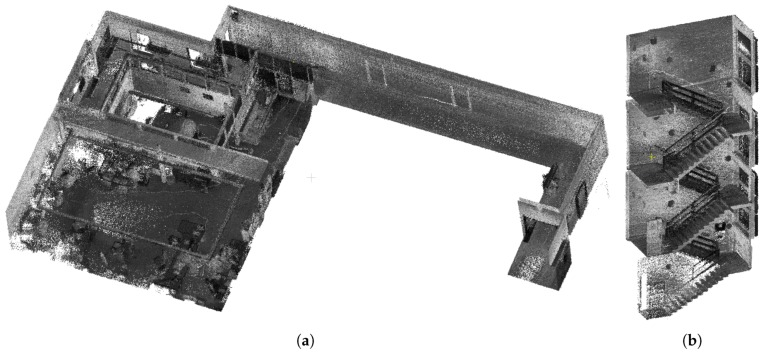
The example of resulting models of indoor mapping. The office environment (**a**) and the staircase (**b**) were captured by a human carrying our 4RECON backpack. The data acquisition process took 3 and 2 min, respectively.

The resulting model has to be dense enough, so that all important objects such as furniture and other inventory can be recognized and distinguished. This is the typical issue of existing solutions such as ZEB-1, where no LiDAR intensity readings are available. Therefore, our solution relies on Velodyne LiDARs, which provide a huge amount of data and the resulting models are dense (see examples in Figure 2). It also provides the laser intensity readings, which do not depend on the lighting conditions, contrary to camera-aided solutions. Moreover, we propose laser intensity normalization, which increases the recognizability of the objects since the laser intensity readings cannot be considered as the “color” of the object as it depends on the range of measurement, the angle of incidence, and the emitted energy.

Some of the existing solutions are not comfortable enough to use. According to practical experience of the operators, handheld solutions such as ZEB are physically difficult to operate for a longer period of time since the mapping head weighs approximately 0.4–1 kg, and it has to be carried or swept by hand.

The final requirement is an affordable price. We use Velodyne VLP-16 scanners, which are relatively cheap in comparison to the other LiDAR solutions, and a universal IMU (Inertial Measurement Unit) solution, which can be upgraded by a dual antenna and therefore reused in the outdoor environment where GNSS (Global Navigation Satellite System) is available.

The contributions of this paper can be summarized as the proposal of a LiDAR mapping solution with the following characteristics:

- It is capable of both small indoor and large open outdoor environments mapping, georeferencing and sufficient precision in the order of centimeters. These abilities are evaluated using multiple datasets.
- It benefits from a synchronized and calibrated dual LiDAR scanner, which significantly increases field of view. Both scanners are used for both odometry estimation and 3D model reconstruction, which enables scanning of small environments, narrow corridors, staircases, etc.
- It provides the ability to recognize objects in the map due to sufficient point density and our novel intensity normalization for the measurements from an arbitrary range.

We also performed a precise evaluation and comparison of our previously proposed point cloud registration method CLS (Collar Line Segments) with state-of-the-art approach LOAM (LiDAR Odometry and Mapping), which has not yet been published. Moreover, we upgraded our CLS method with automatic overlap estimation for better registration flexibility.

## 2. Related Work

LiDAR based systems for indoor and outdoor mapping are not a brand new tool in the geospatial community. Demand for such solutions drives—among other applications, such as autonomous driving—the development of basic algorithms for LiDAR data processing, point cloud registration, etc., as the essential parts of more complex SLAM (Simultaneous Localization and Mapping) methods (a summary can be found in [2]).

Table 1 contains an overview of the existing LiDAR mapping solutions that are related to our work. All such solutions have to solve several typical issues. Besides the construction of hardware mount itself (e.g., a backpack or a drone), the data from multiple sensors have to be synchronized properly, etc. However, the key issue is the software component for odometry estimation—i.e., estimation of the trajectory and the movement of the sensory platform. This is essential for correct alignment of laser measurements into a consistent and precise 3D model. Although there are already numerous methods providing solutions within a certain level of precision for certain types of LiDAR sensors, precise odometry estimation is still an open question.

One of the state-of-the-art methods, performing quite well for both the 3D LiDARs (as Velodyne) and also the 2D rangefinders (as continuously spinning or sweeping Hokuyo LiDAR), is LOAM (LiDAR Odometry And Mapping) [19]. There are also visually [20] or depth enhanced [21] versions where the odometry estimation is supplemented by a RGB camera or a depth sensor (such as Kinect or Asus Xtion), respectively. This whole group can be considered as *feature-based* methods, since, from the original point cloud, only the edge and the plane key points are preserved. These are used for geometrical registration of the current frame within the map and also for building the map itself continuously. Based on the impressive results presented, LOAM method was our first candidate for odometry estimation in our backpack solution. However, our experiments on KITTI Odometry [22] dataset presented later on in Section 4.1 will show that our previously published method CLS (Collar Line Segments) [23] outperforms LOAM in terms of accuracy—the error is lowered from 2.9 cm to 1.7 cm per 1 m of elapsed trajectory.

Another solution for odometry estimation, developed and published by Bosse and Zlot [6] in 2009, is designed for continuously spinning 2D LiDAR rangefinder. After three years, this approach was modified and integrated into the prototype of *Zebedee* [5] mobile mapping application which eventually evolved into ZEB products (in Table 1) of GeoSLAM company [3]. These products are probably the most related to our solution in terms of pricing (ZEB-REVO including 1 year basic support costs 34,000€) and therefore also in terms of accessibility to small companies.

Bosse and Zlot [6] proposed a *surfel-based* algorithm *Voxel Sweep Match* which works over the space discretized into a 3D voxel grid. The model of the environment consists of a set of surfels—3D ellipsoids representing the local surface information within the voxel. The internal model is updated and new surfels are added after each “sweep” (the half revolution of the spinning LiDAR) is captured. The algorithm works similarly to the well known ICP (Iterative closest point) [24], but instead of point-to-point matching, the surfels matching in 9D space (including the position, and the orientation of the surfels) is used. Beside these matching constraints of neighboring surfels, another constraints ensuring the smoothness and the continuity of the trajectory are added in the form of linear equations to be solved. After the new continuous trajectory estimation, surfel positions are updated, and the process is repeated until convergence.

This first proposal [6] did not reach good precision and the main contribution is the basis for further development and improvements—especially missing global loop closure is the problem, which has been solved in downstream projects: Zebedee [5] solution and probably also in ZEB-1 system. It is likely that ZEB-1 is the evolution of Zebedee, since it shares the same ideas and design, but we cannot say this for sure, since it is a closed proprietary solution. Both Zebedee and ZEB-1 use Hokuyo 2D LiDAR, and instead of a continuously spinning mount, a flexible spring construction is used to extend the rangefinder into the 3D LiDAR. The spring amplifies low frequency smooth sweeping motions, while it cancels high frequency motions (vibrations and shaking), which are undesirable and difficult to estimate in SLAM solutions. Moreover, an IMU unit was added in order to estimate quick swinging motion and provide additional constraints for optimization.

**Figure 3:**
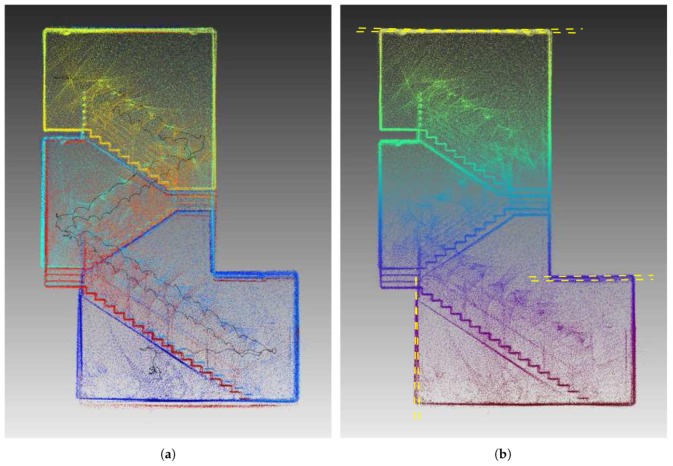
“Double walls” error in the reconstruction of Zebedee [5]. The wall and the ceiling appears twice in the reconstruction, causing an ambiguity. In the solution without loop closure (**a**), the error is quite visible. Double walls are reduced after global loop closure (**b**), but they are still present (highlighted by yellow dashed lines).

Regarding the precision of Zebedee prototype, the accumulated drift for open loop precision causes approximately 10 cm translation and 2° rotation error per minute. This error is significantly reduced by loop closing in a global optimization. The error of the global solution is not published, since the ground truth for experimental dataset was not available. However, the visualization in Figure 3 still shows so-called “dual wall” errors: two instances of the same wall in the same model but at different positions. This ambiguity causes significant problems when the model should be used for further processing (by construction engineers, architects, etc.), and it is our goal to avoid this type of error.

In 2015, the GeoSLAM company released their new alternative version of a handheld LiDAR scanner—ZEB-REVO [7]—where the spring mount was revoked in favor of original continuously spinning design. This update brings better performance in both processing time and accuracy. In addition, the human operator does not have to “whisk” the sensory head in order to correctly capture the whole environment around, as it was required in ZEB-1. However, the weight of the handheld part of the scanner was increased from 0.4 kg (for ZEB-1) to 1 kg, probably due to servo motors and additional electronics. These factors (the necessity to whisk for ZEB-1 and the significant weight for ZEB-REVO) considerably decrease the usage comfort when a larger environment is mapped.

**Figure 4:**
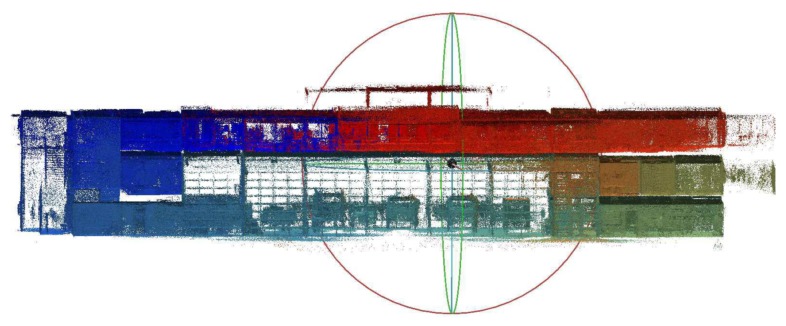
Dataset of indoor office environment for evaluation of ZEB-1 scanner [4]. In the experiment, 3.8 cm error of corner-to-corner average distances within the rooms was achieved.

Since the ZEB products are closed and they are using proprietary software, it is not clear how the 3D map is actually built. Fortunately, there are at least several works published, where the quality of the resulting model was evaluated. The evaluation of ZEB-REVO in an underground quarry [8] reported point precision (in terms of the distance to the best fitting plane for given surface) around 3 cm. In an experiment within a small office environment [25], 22 test planes were selected from the 3D model built by ZEB-REVO. Using the same evaluation, the standard deviation of the point to best fitting-plane distance reached 11 mm. However, these evaluations do not say much about the precision of the whole model and reflect only the local precision. Another work evaluated ZEB-1 [4] by comparison with measurements obtained by a precise terrestrial laser (Leica C10) as the ground truth. For a small indoor environment in Figure 4, the difference in corner-to-corner distances were up to 3.8 cm, and the difference between real and estimated area floor reached 0.4 m^2^. These numbers are consistent with specified positional accuracy between 3–30 cm after 10 min scanning process in user guides [7]. The density of 1000–18,000 points/m^2^ was observed in the point cloud model generated by ZEB-1 which represents an average distance of 0.8–3 cm between the points.

When using Zebedee, ZEB-1 or ZEB-REVO, the user has to follow certain guidelines and also be aware of the limitations of these products [5,7]. When using Zebedee or ZEB-1 with head mounted on the flexible spring, the user has to keep the sensor in the movement by constant “whisking” or somehow changing the accelerations all the time what could be uncomfortable or inconvenient in many cases. The absence of the swinging motion would degrade the sensor back into an 2D rangefinder and could cause a serious error. In addition, the sensors are sensitive to motions in the scene (people, animals, etc.) and the operator has to preserve the overlap between the current and previous measurements—e.g., by walking backwards when leaving a room or traversing doors, keeping a slow pace, etc., since the sensors observe only the environment in front of the operator.

Moreover, there are certain so called “ill” environments or situations when ZEB solutions are failing—especially featureless and empty spaces, where SLAM solutions are failing in general, and the only solution is the augmentation of the scene by additional obstacles, boxes, etc. Optimal results can be obtained when the obstacles or featuring objects are within 15–20 m range for outdoor. This is a significant limitation for vast open environments.

Other mobile backpack solutions for the LiDAR mapping can be divided into two groups: fully commercial, such as Leica Pegasus [11], Viametris bMS3D [13,14], Robin backpack [15], or GreenValley LiBackpack [9], and research projects, such as Akhka Backpack [17]. Basic properties of these solutions are summarized in Table 1. The most significant drawback of these solutions is their high price: 150,000 € for Pegasus, 220,000 € for Robin, and 60,000 € for GreenValley backpack (without GNSS upgrade), which makes them too expensive and inaccessible for small businesses. In comparison, the total cost of HW components in our solution is around 17,500 €. For the whole product (including SW development, support, etc.) we expect the price to increase approximately twofold, which brings us much closer to ZEB scanners. Another disadvantage of these backpack solutions (at least for Leica Pegasus and Robin backpacks) is their high dependency on GNSS, so the quality of mapping drops when the signal of satellites is poor or not available.

Leica, Viametris, 3D Laser Mapping, and GreenValley companies naturally did not publish how their solutions estimate the odometry and the alignment of LiDAR data into 3D model. We know that these systems use GNSS/INS aiding in order to improve the precision. According to the documentation [11], Leica Pegasus is able to achieve up to 5 cm precision after 10 min walk, when GNSS is available and 5–50 cm without GNSS aiding. It uses 2 Velodyne LiDAR scanners as a source of 3D data and an additional set of five high-resolution cameras. Potential problems for small rooms, staircases and featureless environments are reported in the documentation. Independent evaluation has been performed in small (20 m length) underground medieval stronghold [12], where average error of 4.2 cm is reported when the model is compared with terrestrial LiDAR reference. There is not much information published regarding price or precision of Viametris backpack. However, up to 5 cm accuracy is reported when reasonable satellite reception is available [13]. The Robin backpack [15] for outdoor mapping depends on precise GNSS/INS (Inertial Navigation System) with dual antenna, claiming 2 cm positional and 0.03° error. However, the precision of generated models is not specified, and no evaluation papers have been published yet (to our best knowledge). The specification of GreenValley LiBackpack [9] claims ≈5 cm relative accuracy of the system.

LiBackpack can be considered as the backpack solution most similar to ours—in terms of price, sensors, and accuracy. However, according to the information given to us by GreenValley company, their solution uses Velodyne scanners separately—one scanner is used for the odometry estimation using SLAM, and the second one for the 3D reconstruction. We find it unfortunate, because the full potential of data is not utilized. In the solution proposed in this paper, both scanners are synchronized and extrinsically calibrated—mutual 6 DoF (Degrees of Freedom) pose is estimated. This makes it possible to use both sensors in both tasks—SLAM and building the 3D model.

Akhka mapping backpack [17,18,26,27] was developed by Finnish Geospatial Research Institute and Aalto University. It deploys Faro Focus LiDAR and depends on the precise Novatel Flexpak6 GNSS-IMU solution. When mapping the environments with wrong GNSS reception, the scans are roughly aligned by IMU within small time windows—segments. Afterwards, ICP is used for registration of these segments. During the experiment in a river channel, RMSE (root mean square error) of 3.6 cm was measured at reference positions. During the mapping of a forest environment, the average misalignment increased to 8.7 cm.

Google released their SLAM software Cartographer [28] for online building 2D floor plans using LiDAR rangefinders. It uses efficient probability 2D occupancy grid (5 cm resolution) as a map representation enabling fast registration and robust loop closure. Google also claims the ability to produce full 3D maps, however, the results reported are not that appealing [29]. As far as we know, no seriously evaluated deployment has been published so far.

The idea behind the well-known KinectFusion [30] project for processing RGB-D data drove the development of a new solution for LiDAR odometry estimation called IMLS-SLAM [31]. Instead of typical scan-to-scan matching and registration, the target LiDAR scan is transformed into implicit surface representation denoted as IMLS surface (Implicit Moving Least Square) originally proposed by [32]. The source frames are registered against these implicit surfaces following the scan-to-model strategy. This work also provides mathematical background for solving such a task as a least-square optimization problem. On average, their method achieved 0.69 cm drift after 1 m of elapsed trajectory.

Droeschel et al. [33] proposed a hierarchical pose graph structure for online mapping and odometry estimation. They split each frame into scan lines (slices of the Velodyne LiDAR frame with 1.33 ms duration), while they also group neighboring frames into local optimization windows. Therefore, there are 3 types of nodes within the graph: map nodes representing local windows on the highest level, scan nodes representing Velodyne LiDAR frames (360° revolution), and the scan line nodes on the lowest level. The surfel based registration is performed only among the frames within the local window (forming edges between map and scan nodes) and among whole local windows (producing edges among map nodes). The global optimization produces a continuous time trajectory, where the transformation is assigned to each scan line by cubic B-spline interpolation. Therefore, the scans, the pose graph, and the trajectory are iteratively refined. Unfortunately, the paper does not provide the precise evaluation of this method. The visualizations show that the method reduces the thickness of the walls and so-called “double wall” effect in comparison with previous approach without hierarchical structure [34].

We also experimented with a similar hierarchical approach in our SLAM system. The main motivation was to make the process more time-efficient. Eventually, we rejected this idea, since the errors of frame-to-frame registrations, which were introduced into the local map, made the registrations among local maps quite inaccurate.

Mendes et al. [35] decided to run a simple ICP frame-to-frame registration for the stream of LiDAR scans. Instead of registering the consecutive frames together, the current frame is aligned within the local map consisting of last few keyframes. When the overlap (given by the point matching in ICP algorithm) drops under a certain level, the current scan is labeled as a new keyframe and it is added to the local map. The old keyframes (dropped from the local map) are preserved for the loop detection and closure.

Besides the geometrical accuracy of the model, there are also other quality aspects to consider when creating a LiDAR mapping solution, e.g., the point cloud density and especially the ability of so-called “recognizability” of various objects in the map. The consumer of the point cloud model (engineer, architect, geodesist, etc.) has to be able to recognize furniture, surface borders, and in some cases also writings, symbols or the texture of the surface. For this task, the color or at least the intensities have to be correctly introduced into the model. In the previously described solutions of LiDAR mapping, this information is missing (e.g., ZEB-1, basic ZEB-REVO) or introduced by additional RGB camera (e.g., ZEB-REVO [36]). In solutions based on the terrestrial laser scanner or Velodyne LiDARs, the intensity of laser return is used directly to color the points in the model.

**Figure 5:**
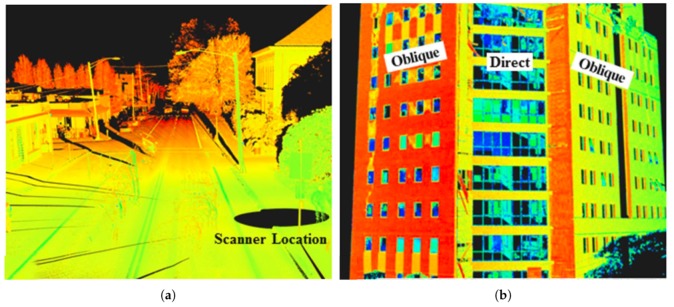
The dependency of laser intensity readings (weak readings in red, strong in green) on the measurement range (**a**) and the angle of incidence (**b**) [37].

Since we want to keep our solution simple and cheap and preserve the invariance to lightning conditions, we decided to “color” our models with LiDAR intensities. However, keeping these raw intensities would cause unwanted artifacts. As was described in previous works [37,38,39], the reflectivity of the surface, which we want to capture, is not the only factor affecting these intensity values. The measured intensity depends also on the incidence angle of the laser ray, distance from the sensor (see Figure 5), power of laser transmitter, and, in some cases, also on the atmospheric influences (e.g., fog, dust, and smog). These works addressed the problem providing models and closed form solutions. However, these methods are valid only for large-distance measurements (at least tens of meters) and therefore they are not suitable for typical indoor or smaller outdoor environments, which we need to address. Hence, we propose a novel probabilistic method for LiDAR intensities normalization which is scalable and capable of processing near-distance measurements.

## 3. Design of the Laser Mapping Backpack

This section consists of two main parts: First, the hardware design concepts are introduced. Then, the software solutions dealing with calibration, precise odometry estimation, alignment and intensity normalization are presented.

**Figure 6:**
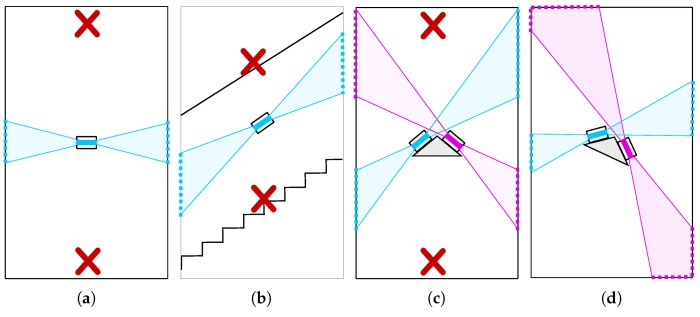

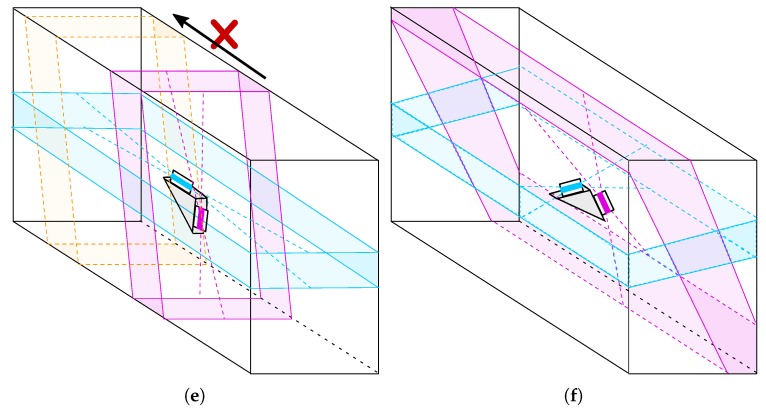
Various configurations of LiDAR scanners in worst case scenarios we have encountered in our experiments: narrow corridor (**a**,**c**) and staircase (**b**). The field of view (30° for Velodyne Puck) is displayed in color. When only single LiDAR (**a**) was used, the scans did not contain 3D information of the floor or the ceiling (red cross). The situation was not improved when the scanner is tilted because of failing in, e.g., staircases (**b**). When we added a second LiDAR, our tiled asymmetrical configuration (**d**) provides better top–bottom and left–right observation than the symmetrical one (**c**). Moreover, when the LiDARs are aligned in direction of movement (**e**), there is no overlap between current (violet) and future (yellow) frame, leading to lower accuracy. In our solution (**f**), the LiDARs are aligned perpendicularly to the walking direction solving all mentioned issues.

The design of our solution follows the requirements elaborated in Section 1. They have been carefully formulated and discussed with experts in the field of geodesy and geospatial data processing. Besides the essential goal of reliable 3D reconstruction performed automatically, which is demonstrated in the following section, the proposed solution does the following:

- It fulfils the requirements for precision of the model up to 5 cm. Thanks to the robust loop closure, ambiguities (e.g., “double wall” effects) are avoided.
- The system is comfortable to use and it is as mobile as possible. The backpack weighs 9 kg (plus 1.4 kg for the optional dual antenna extension), and it is easy to carry around various environments including stairs, narrow corridors, rugged terrain, etc.
- The pair of synchronized and calibrated Velodyne LiDARS increases the field of view (FOV) and enables mapping of small rooms, narrow corridors, staircases, etc. (see Figure 6) without the need for special guidelines for scanning process.
- The data acquisition process is fast with verification of data completeness. There are no special guidelines for the scanning process (comparing to the requirements of ZEB) and the operator is required only to visit all places to be captured in a normal pace. Moreover, captured data are visualized online at the mobile device (smartphone, tablet) for operator to see whether everything is captured correctly.
- Since we are using long range Velodyne LiDAR (compared to simple 2D rangefinders such as Hokuyko or Sick) and optional GNSS support, we provide a universal economically convenient solution for both indoor and outdoor use. For such scenarios, where GNSS is available, final reconstruction is georeferenced—the 3D position in the global geographical frame is assigned to every 3D point in the model.
- The final 3D model is dense and colored by the laser intensity, which is further normalized. This helps distinguishing important objects, inventory, larger texts, signs, and some surface texture properties.

### 3.1. Hardware Description

**Figure 7:**
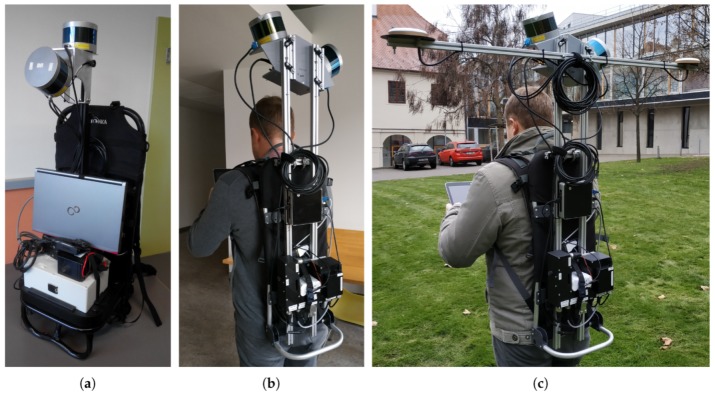
The initial (**a**) and improved (**b**,**c**) prototype of our backpack mapping solution for both indoor (**b**) and outdoor (**c**) use. The removable dual GNSS antenna provides precise heading information, aiding for outdoor odometry estimation and also georeferencing of the resulting 3D point cloud model. It should be noted that the position of LiDAR scanners is different in the initial and the later solution. This is elaborated on in the next section.

The core of our backpack, in Figure 7, is the pair of Velodyne LiDAR [40] scanners VLP-16 (Pucks). Each of them contains 16 laser transmitter–receiver pairs, which are reflected into the environment by a rotating mirror with 10 Hz frequency. This frequency can be decreased or increased up to 20 Hz. However, frequency higher than 10 Hz causes serious undesirable vibration of the sensor, which makes precise odometry estimation impossible. The rotation gives the sensor 360° horizontal FOV with 0.2° horizontal resolution. Vertically, the laser beams are evenly distributed with 2° resolution covering 30° vertical FOV. Each of the scanners weighs 830 g and is considered to be a hybrid solid state LiDAR, since there are no outer moving parts. This type of scanner is able to reach 100 m range with precision around 2 cm. As mentioned above, Velodyne scanners provide also values of intensity readings, which corresponds to the surface reflectivity.

**Figure 8:**
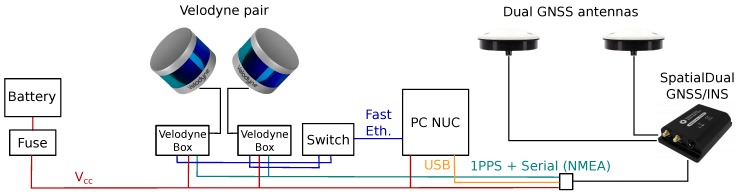
Components of the system and the connections. Each Velodyne scanner is connected via a custom wiring “box” requiring power supply (red wires), 1PPS and NMEA synchronization (green) and Fast Ethernet (blue) connection with computer (PC NUC in our case).

As the aiding sensor, the GNSS/INS (Inertial Navigation System) Advanced Navigation SpatialDual (https://www.advancednavigation.com/product/spatial-dual) is deployed. It integrates multiple sensors such as accelerometers, gyroscopes, magnetometer, pressure sensor, and most importantly—the dual-antenna GNSS subsystem providing reliable heading information. With RTK (Real Time Kinematics) or PPK (Post-Processed Kinematics) corrections, the system should provide 8 mm horizontal and 15 mm vertical positional accuracy, and 0.03° and 0.06° orientation precision in terms of roll/pitch and heading angle, respectively. Precise heading information is provided by a dual antenna solution and therefore it is only available outdoors. This limitation also holds for positional data. For indoor scenarios, only roll and pitch angles are reliable and they are relevant for horizontal alignment. The unit weighs 285 g and besides the 6 DoF (six Degrees of Freedom including 3D position and rotation) pose estimation it also provides 1PPS (Pulse Per Second) and NMEA messages for precise synchronization of both Velodyne LiDAR scanners. The details regarding wiring the components can be found in Figure 8.

The rest of the hardware is responsible for controlling the data acquisition and storing the data (Intel NUC Mini PC), and powering all the components with small Li-Ion battery with capacity 10,400 mAh lasting approximately 2 h.

### 3.2. Dual LiDAR System

During the experiments, we discovered that the limited (30°) horizontal field of view is not an issue for large open spaces. However, when the space is getting smaller and the environment shrinks (e.g., corridors narrower than 2 m), such a field of view causes serious problems, leading to poor accuracy or even total failures of the SLAM system. The worst cases and our solutions are displayed in Figure 6. We experimentally discovered that we need at least two synchronized Velodyne Puck scanners to provide a robust solution that covers both the floor/ceiling and the walls, even in small or narrow rooms.

To achieve good accuracy and to cover the environment, the scanners are mounted perpendicular to the direction of the operator movement—one in horizontal and second in vertical orientation, as displayed in Figure 6f and Figure 7b,c. All other configurations (e.g., Configuration e.) in our initial prototype in Figure 7a were not able to capture both horizontal and vertical properties of the environment, or did not provide a large coverage necessary for precise pose estimation.

### 3.3. Calibration of the Sensors

To leverage the full potential of using two Velodyne LiDARs, these scanners have to be properly synchronized and calibrated. As mentioned above, the sensors are synchronized via NMEA messages (GPS communication protocol) and 1PPS (Pulse Per Second) signal provided by SpatialDual inertial navigation system. Sufficient intrinsic calibration parameters of LiDAR scanners themselves (corrections) are provided by Velodyne company and processed by the driver (in ROS Velodyne package).

**Figure 9:**
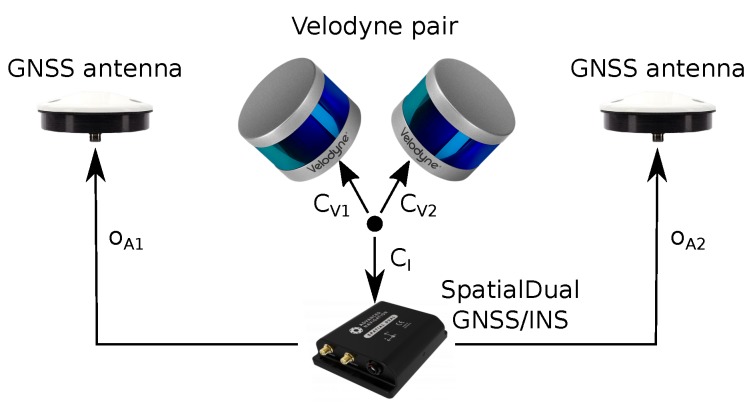
Extrinsic calibration required in our system. The mutual positions between the Velodyne scanners and the GNSS/INS unit are computed. The offsets ${\vec{o}}_{A1},{\vec{o}}_{A2}$ of the antennas are tape measured.

**Figure 10:**
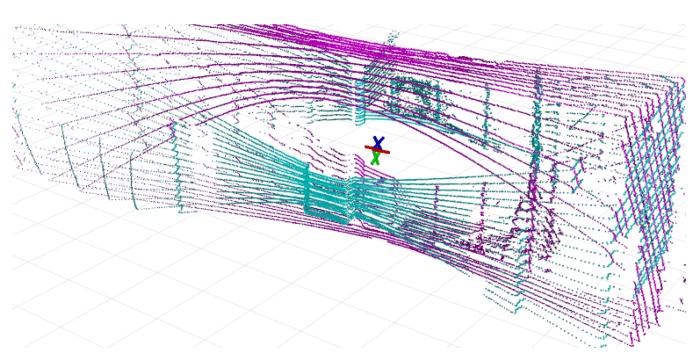
Two Velodyne LiDAR frames aligned into the single *multiframe*. This data association requires time synchronization and precise extrinsic calibration of laser scanners.

Therefore, the task to solve is the estimation of extrinsic calibration parameters in terms of relative 6DoF pose estimation for both laser scanners $C_{V1},C_{V2}$ and INS sensor $C_{I}$ in Figure 9. First, the transformation between the scanners is computed. To do so, two 3D maps of a large indoor space (a large lecture hall in our case) were built by the scanners separately using our previously published method [23]. These two 3D maps are ICP aligned. The resulting 3D geometrical transformation represents mutual position of the sensors $C_{V1}^{-1}*C_{V2}$ and also the alignment of laser data they provide as presented in Figure 10. Since we are interested only in relative transformations between the sensors, the origin can be arbitrarily defined, e.g., as the position of the first Velodyne and $C_{V1}=I$. A single frame point cloud consists of multiple (two in our case) synchronized LiDAR frames and therefore it will be denoted as the *multiframe*.

To be able to use data provided by the INS system, an extrinsic calibration $C_{I}$ between the laser scanners and the INS sensor needs to be estimated. All sensors are fixed on the custom made aluminum mount and therefore the translation parameters can be found in the blueprints of the mount or can be measured with millimeter precision. However, mutual rotation has to be estimated more precisely, because just a fraction of degree misalignment would cause serious errors for long range laser measurements.

We found that the rotation parameters as the transformation between the floor normal vector ${\vec{n}}_{i}$ in the point cloud data and the gravity vector ${\vec{g}}_{i}$ provided by the INS sensor, since these vectors should be aligned. Points of the floor are selected manually and the normal of the best fitting plane is computed. This can be performed in arbitrary software for visualization and processing of the point clouds—CloudCompare (https://www.danielgm.net/cc/) in our case. We performed multiple measurements for different inclines of the backpack in the indoor corridor with a perfectly straight floor. The final rotation $R_{C_{I}}$ between the Velodynes and INS sensor was estimated by SVD (Singular value Decomposition) [41] (Equation (2)) of covariance matrix A of these 3D vector pairs (Equation (1)) (floor normal and the gravity). Multiplication with matrix E (Equation (5)) solves the ambiguity between right/left hand rotation—we always compute right-hand representation. Equations (1)–(5) are based on the work [41].

(1) $$\begin{array}{cc} A & =\sum_{i}{{\vec{n}}_{i}}^{T}\cdot {\vec{g}}_{i} \end{array}$$

(2) $$\begin{array}{cc} U\Sigma V^{*} & =A \end{array}$$

(3) $$\begin{array}{cc} e & =\left\{\begin{array}{cc} 1, & \mathrm{if}\;|VU^{T}|\geq 0\\ -1, & \mathrm{otherwise} \end{array}\right. \end{array}$$

(4) $$\begin{array}{cc} E & =\left[\begin{array}{ccc} 1 & 0 & 0\\ 0 & 1 & 0\\ 0 & 0 & e \end{array}\right] \end{array}$$

(5) $$\begin{array}{cc} R_{C_{I}} & =VEU \end{array}$$

### 3.4. Point Cloud Registration

The core element of the software part is the alignment of the point cloud data into a 3D map of the environment. There are multiple state-of-the-art approaches for point cloud registration and odometry estimation, including our previously published approach Collar Line Segments [23]. We compared our approach with LOAM [19] algorithm, using the implementation available. The results of this experiment are presented in Table 2, which shows the superior accuracy of our method, thus CLS was a natural choice for our mapping backpack solution.

**Figure 11:**
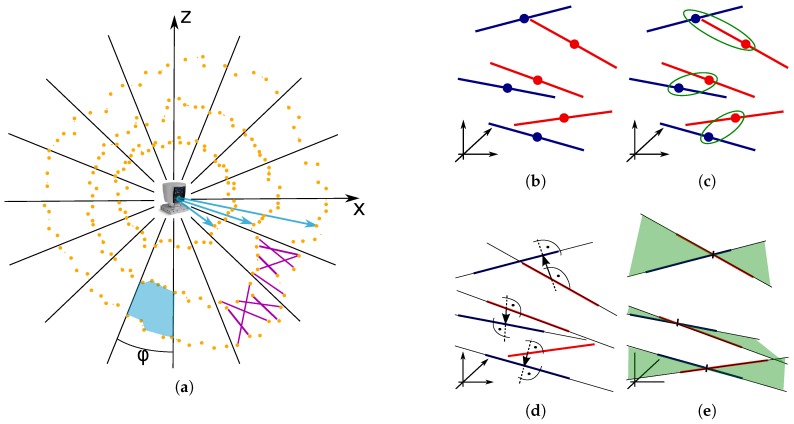
The sampling of Velodyne point cloud by the Collar Line Segments (CLS) (**a**). The segments (purple) are randomly generated within the polar bin (blue polygon) of azimuthal resolution ϕ. The registration process (**b**–**e**) transforms the line segments of the target point cloud (red lines) to fit the lines of the source cloud (blue). First, the lines are matched by Euclidean distance of midpoints (**c**); then, the segments are extended into infinite lines and the vectors between closest points are found (**d**); and, finally, they are used to estimate the transformation that fits the matching lines into common planes (green in (**e**)).

The basic idea of the CLS method is to overcome the data sparsity of 3D LiDAR scanner (e.g., Velodyne) by sampling the data by line segments. The points captured by individual laser beams form so called “ring” structures displayed in Figure 11a. There is a large empty space between these rings and while moving, same places of the scene are not repeatedly scanned, valid matches are missing and the closest point approaches (e.g., ICP) are not applicable. By using CLS, the space between the rings is also covered and correct matching of structures in the LiDAR frames is enabled.

The environment in the field of view is represented by the set of CLS line segments. They are randomly generated between the neighboring ring points within the azimuthal bin as described in Figure 11a. Since we are using two LiDAR scanners, collar line segments are generated for the scans of each sensor individually. Using the transformation established by extrinsic calibration described in Section 3.3, line segments are transformed and joined into the single set for each multiframe.

After the sampling is done, matching of the closest line segments is performed. The line segments are extended into the infinite lines, and the closest points between matching lines are used for direct estimation of translation. SVD [41] is used again for estimation of rotation parameters in the same manner, as described in Section 3.3. This description is only a brief introduction to the CLS method and more information can be found in our previous publication [23].

### 3.5. Overlap Estimation

This work provides a novel solution for automatic estimation of the core parameter of the CLS approach. Before the transformation is estimated, invalid matches must be discarded. In our previous work, this was done by a simple distance thresholding, or by keeping a certain portion of matches (e.g., 50%). However, using a constant threshold or portion value is not flexible enough. It can cause significant registration misalignments, when invalid matches are used, or insufficient convergence when the valid matches are ignored.

Assuming that an initial coarse alignment is known, we are able to estimate *the overlap* between these frames and use this value as the portion of matches to keep (e.g., for 30% overlap, 30% of best matches are kept). This solution adapts to the specific situation of each pair of LiDAR frames to be registered and leads to a significantly better precision.

**Figure 12:**
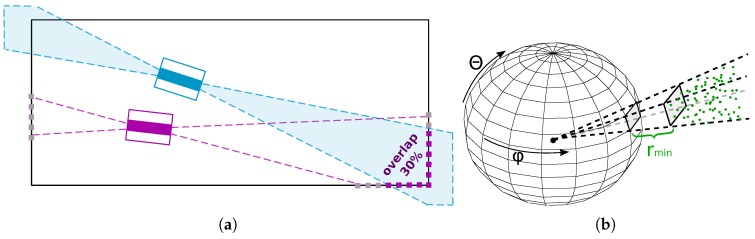
The overlap (**a**) between the source (blue) and the target (purple) LiDAR frame. In this case, approximately 30% of source points are within the view volume of target frame. The view volume can be effectively represented by *spherical z-buffer* (**b**) where range information (minimum in this case) or the information regarding empty space within the spherical grid is stored.

**Figure 13:**
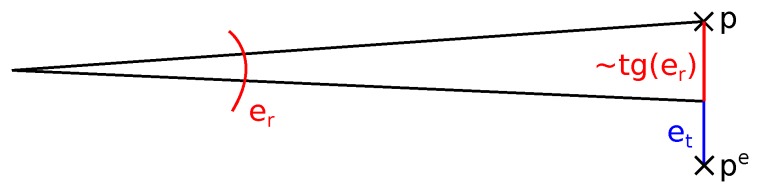
The error of measurement (Euclidean distance between points *p* and $p^{e}$) can be split into rotation $e^{r}$ and translation $e^{t}$ part. The impact of rotation error $2\cdot \mathrm{tg}(e_{r}/2)$ can be simplified to $\mathrm{tg}(e_{r})$ due to near linear properties of tangent function for small angles.

The overlap value (Figure 12a) is effectively estimated by *spherical z-buffer* structure [42] in Figure 12b. First, the target cloud is transformed into the source cloud coordinate frame and the [x,y,z] coordinates of all the points are transformed to spherical coordinates ϕ,θ,r (polar angle, elevation angle, and range). Each spherical bin of the z-buffer is assigned with minimal range value from the source point cloud. The minimal value is chosen since unwanted reflections sometimes cause invalid long range measurements and therefore there is the best chance that the minimum range measurement is valid. Then, all the points of target point cloud (also transformed to spherical coordinates) with range below the value in z-buffer (including certain tolerance) are considered to be overlapping points and the ratio to all the points is considered to be the *overlap* value. More formally, if the point *p* with range $p_{r}$ within the spherical bin *i* fulfills the requirement

(6) $$p_{r}<r_{min}^{i}\cdot t_{r}+t_{a},$$

it is considered to be a part of the overlap. Value $r_{m}in^{i}$ denotes the minimal range value stored within the spherical bin. Absolute $t_{a}$ and relative $t_{r}$ tolerance values represent the acceptable translation and rotation error. Especially the error of rotation causes larger displacements for larger ranges. Equation (6) follows our *error model*, where the error *e* is the distance between precise point coordinates *p* (which are unknown) and known erroneous coordinates $p^{e}$ which can be approximately estimated as:

(7) $$e=|p-p^{e}|=p_{r}^{e}\cdot \mathrm{tg}\left(e_{r}\right)+e_{t},$$

where $e_{r}$ represents rotation, $e_{t}$ is the translation error, and $p_{r}^{e}$ is the range of the erroneous point (see also Figure 13). In our experiments, we used the tolerance values $t_{r}=0.1$ and $t_{a}=0.3$ for the overlap estimation. This allows rotation error $e_{r}$ approximately 5° and translation error 30 cm for the initial coarse transformation between the scans.

### 3.6. Rolling Shutter Corrections

**Figure 14:**
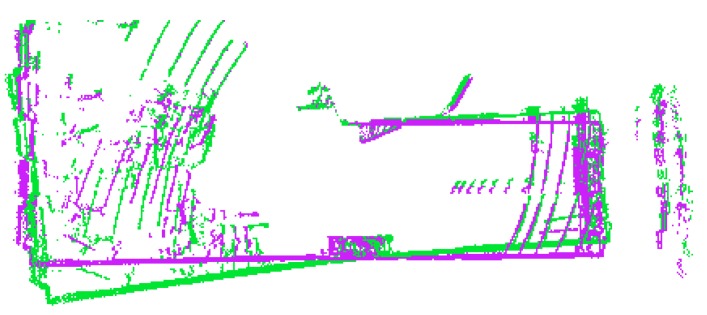
Example of a LiDAR frame distorted by the rolling shutter effect when the operator with mapping backpack was turning around (green) and the corrected frame (purple). This is the top view and the distortion is mostly visible on the “bent” green wall at the bottom of this picture.

As mentioned in the description of Velodyne sensor, spinning frequency is approximately 10 Hz which leads to 100 ms duration of a single LiDAR scan acquisition. This is a relatively long time when significant movement is assumed. Large translation in the case of fast vehicles or possible fast rotations in case of human carrier can cause distortions in LiDAR frame displayed in Figure 14. We denote this effect as *rolling shutter* because it resembles rolling shutter distortion of optical sensors.

This means that the LiDAR data cannot only be rigidly transformed, but a continuous transformation needs to be applied or at least approximated. The single Velodyne Puck frame consists of approximately 75 packets, each carrying a slice of the frame. Slices are evenly distributed in both time and space. Thus, for each *i*th frame, we compute the relative transformation $T_{i\to j}$ that occurred during the acquisition of the current frame using the global position $P_{i}$ of the current frame and the pose $P_{i+1}$ of the next one as:

(8) $$T_{i\to j}=P_{i}^{-1}\cdot P_{i+1}.$$

The correction for each slice is estimated by interpolation of this transformation. The translation parts are interpolated linearly and, for the rotations, Spherical Linear Interpolation (SLERP) [43] over quaternion representation is used. For the first slice, zero transformation is estimated and the last one is transformed by $T_{i\to j}$.

### 3.7. Pose Graph Construction and Optimization

**Figure 15:**
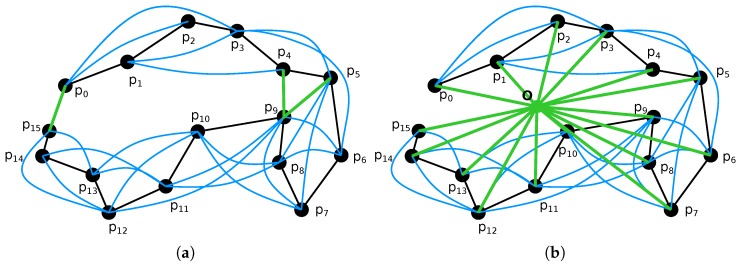
Pose graph as the output of point cloud registration and the input of SLAM optimization. The goal is to estimate 6DoF poses $P_{1},P_{2},\ldots ,P_{N}$ of graph nodes (vertices) $p_{1},p_{2},\ldots ,p_{1}5$ in the trajectory. The edges represent the transformations between LiDAR frames for given nodes estimated by point cloud registration. Black edges represent transformations between consequent frames, blue edges are for transformations within a certain neighborhood (maximum distance of three frames in this example) and the green edges (in (**a**)) represent visual loops of revisited places detected by a significant overlap between the given frames. When GNSS subsystem is available (**b**), additional visual loops are introduced as transformations from the origin O of some local geodetic (orthogonal NED) coordinate frame.

The proposed CLS method for point cloud alignment can only provide consecutive frame-to-frame registration. However, since each registration is burdened by a small error, after some time, the accumulated error (*drift*) is no longer acceptable. To reduce this drift and also to close loops of revisited places, we propose an iterative process of *progressive pose graph* construction and optimization. The key idea of this algorithm is progressive refinement of odometry estimation from local precision within small time window to global precision across the whole model. This iterative method is described in Figure 15 and more formally in Algorithm 1.

First, only consecutive frames (within neighborhood of size 1) are registered, and then the neighborhood is gradually enlarged (size *d* in Algorithm 1, step 1) until it covers all *N* frames. CLS registration is performed for each pair (*i*th and *j*th frame) within the current neighborhood where a significant overlap is found and then efficient pose graph optimization using SLAM++ framework [44] is performed. Modulo operator in Step 3 reflects the fact that we assume a circular trajectory. This assumption of beginning and ending the data acquisition process at the same place is common also for other similar solutions (ZEB-1, ZEB-REVO, etc.) [7]. It helps the system to identify at least one visual loop that guarantees reasonable results from the global SLAM-based optimization.

**Algorithm 1** Progressive refinement of 6DoF poses ${\left\{P_{i}\right\}}_{i=1}^{N}$ for sequence of frames ${\left\{f_{i}\right\}}_{i=1}^{N}$ by optimizing pose graph G.
 1:
**for** d=2 **to** $\frac{N}{2}$ **do**
 2:  **for** i=1 **to** *N* **do** 3:   j:=(i+d)modN 4:   $T_{i\to j}:=P_{j}^{-1}\cdot P_{i}$ 5:   $o_{ij}:=\mathrm{OVERLAP}\left(f_{i},f_{j},T_{i\to j}\right)$ 6:   **if** $o_{ij}>t_{o}$ **then** 7:    $T_{i\to j},e:=\mathrm{CLSREGISTARTION}\left(f_{i},f_{j},T_{i\to j},o_{ij}\right)$ 8:    **if** $e\leq \mathrm{MEDIANRANGE}\left(f_{i}\right)\cdot t_{r}+t_{a}$ **then** 9:     $G:=G\cup \{\mathrm{EDGE}\left(i,j,T_{i\to j}\right)\}$10:    **end if**11:   **end if**12:  **end for**13:  $P_{1},P_{2},\ldots ,P_{N}=\mathrm{OPTIMIZE}\left(G\right)$14:
**end for**
15:
**return** $P_{1},P_{2},\ldots ,P_{N}$



Before a pair of frames is registered, the presence of overlap larger than $t_{o}$ is verified (Line 5 in Algorithm 1) in order to preserve the registration stability. We used minimal 0.5 overlap in our experiments. This also plays the role of visual loop detection every time a place is revisited.

Moreover, after the CLS registration is performed, we verify the result of registration (Line 8) using the error model described in Equation (7). As the reference range value, we take the median range of the source point cloud. In our experiments, we used tolerance values $t_{r}=0.01$ and $t_{a}=0.05$ representing tolerance of approximately 0.5° in rotation and 5 cm in positional error.

For outdoor mapping, the absolute position and orientation are provided by the GNSS/INS subsystem with PPK (Post Processed Kinematics) corrections. While the global error of these poses is small, relative frame-to-frame error is much larger when compared to the accuracy of pure SLAM solution. Therefore, we combine our SLAM (in the same way as described above) with additional edges in the pose graph representing the global position in some geodetic frame, as shown in Figure 15b.

### 3.8. Pose Graph Verification

After the registration is performed, a new edge is added into the pose graph only if the registration error is below a certain threshold modeled by Equation (7) (Line 8 of Algorithm 1). However, this simple rejection is not robust enough—some registrations are falsely rejected or accepted. After all overlapping frames are registered, additional verification is performed for all edges.

**Figure 16:**
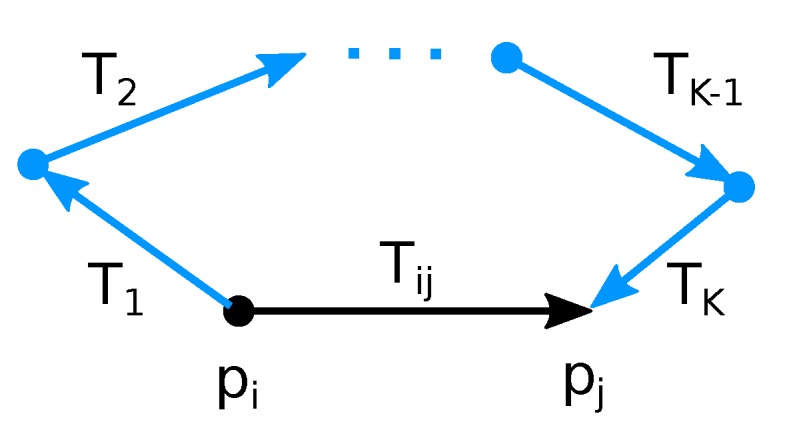
Verification of edge $\left(p_{i},p_{j}\right)$ representing transformation $T_{ij}$ is performed by comparison with transformation $T_{1}\cdot T_{2}\ldots T_{K}$ of alternative path (blue) between *i*th and *j*th node.

Expected transformation $T_{ij}^{e}$ is computed (Equation (9)) using alternative path $T_{1},T_{2},\ldots T_{K-1},T_{K}$, as described in Figure 16. The L2 norm of positional difference between expected transformation $T_{ij}^{e}$ and the transformation $T_{ij}$ found by registration (Equations (10)–(12)) is considered as the error value related to this edge. Note that the positional difference is also affected by the difference in rotation and therefore it is included in this error.

(9) $$\begin{array}{cc} T_{ij}^{e} & =T_{1}\cdot T_{2}\cdot \ldots \cdot T_{K-1}\cdot T_{K} \end{array}$$

(10) $$\begin{array}{cc} {∆}_{ij} & =T_{ij}^{-1}\cdot T_{ij}^{e} \end{array}$$

(11) $$\begin{array}{cc} {∆}_{ij} & =\left[R_{ij}|t_{ij}\right] \end{array}$$

(12) $$\begin{array}{cc} e_{ij} & ={∥t_{ij}∥}_{2} \end{array}$$

For each edge, all alternative paths up to a certain length are found and their errors are estimated. We use paths of length up to 3 as a tradeoff between the time complexity and robustness. An edge is rejected when the median of these error values is below accepted threshold (10 cm in our experiments). This cannot be considered as target error of our reconstruction since the pose graph optimization process further decreases the cumulative error. The whole process is repeated until there is no edge to reject.

### 3.9. Horizontal Alignment of the Indoor Map

**Figure 17:**
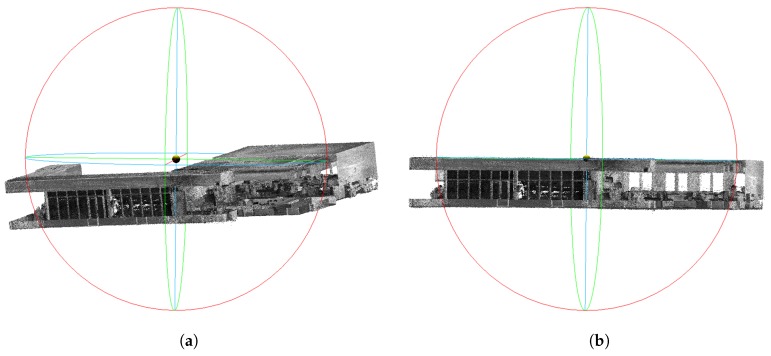
The reconstruction built by our SLAM solution before (**a**) and after (**b**) the alignment of horizontal planes (floor, ceiling, etc.) with XY plane (blue circle).

While, for outdoor environment, the model is georeferenced and aligned with NED geodetic coordinate frame (north, east, and down), there is no such possibility when mapping indoors since the GNSS signal is not available. However, practical indoor applications of our 3D mapping solution require at least horizontal alignment—the alignment of gravity vector with Z-axis and the alignment of straight floors/ceilings with XY-plane in resulting 3D model as Figure 17 shows.

This alignment is possible, since roll and pitch angles are provided by IMU (using measurements by accelerometers and gyroscopes) and extrinsic calibration of Velodyne sensors to the IMU frame $C_{I}$ estimated as described in Section 3.3. The simplest solution would be to use these roll and pitch angles directly to align the LiDAR scans individually and deploy the SLAM only to estimate the remaining parameters (heading and translation). Unfortunately, this is not possible because the accuracy of roll and pitch angles is not sufficient—error in order of degrees happens during the motion. Since our goal is to reduce the cost of our solution, we did not want to use additional expensive hardware. We rather propose an alternative approach to estimate horizontal alignment from these noisy measurements.

We can leverage the fact that there are multiple (thousands) of roll/pitch measurements and only a single transformation for horizontal alignment needs to be computed. First, we are able to split each transformation (for each LiDAR frame) estimated by SLAM into the rotation and the translation

(13) $$P_{SLAM}=\left[R_{SLAM}|t_{SLAM}\right].$$

Our partial goal is to estimate horizontal alignment $A_{h}$ fulfilling Equation (14). The transformation of point cloud data *X* by SLAM rotations $R_{SLAM}$ and horizontal alignment $A_{h}$ is the same, as the transformations of these data by IMU measured rotation $R_{IMU}$ (including the calibration $C_{I}$). In addition, each rotation (SLAM or IMU provided) can be split into roll $R^{R}$, pitch $R^{P}$ and heading $R^{H}$ (Equation (15)). Since the IMU sensor is not able to provide accurate heading information indoors, we supplement the heading $R_{SLAM}^{H}$ estimated by SLAM.

(14) $$\begin{array}{cc} R_{IMU}\cdot C_{I}\cdot X & =A_{h}\cdot R_{SLAM}\cdot X \end{array}$$

(15) $$\begin{array}{cc} R_{SLAM}^{H}\cdot R_{IMU}^{P}\cdot R_{IMU}^{R}\cdot C_{I} & =A_{h}\cdot R_{SLAM} \end{array}$$

(16) $$\begin{array}{cc} A_{h} & =R_{SLAM}^{H}\cdot R_{IMU}^{P}\cdot R_{IMU}^{R}\cdot C_{I}\cdot R_{SLAM}^{-1} \end{array}$$

Using Equation (16), we are able to estimate the (noisy and inaccurate) horizontal alignment $A_{h}$ for each pair of SLAM and IMU provided rotations of the same timestamp. During the mapping, there are usually thousands of these pairs (10 pairs per second) which are synchronized. The precise horizontal alignment is then computed by averaging the quaternions [45] representing noisy partial alignments $A_{h}$.

### 3.10. Intensities Normalization

**Figure 18:**
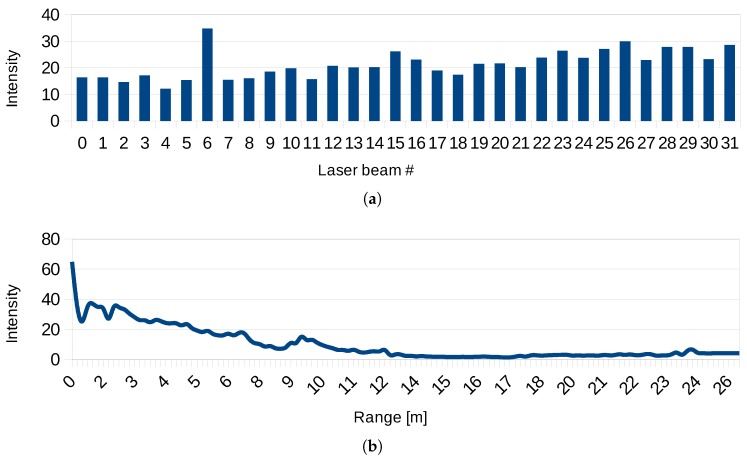

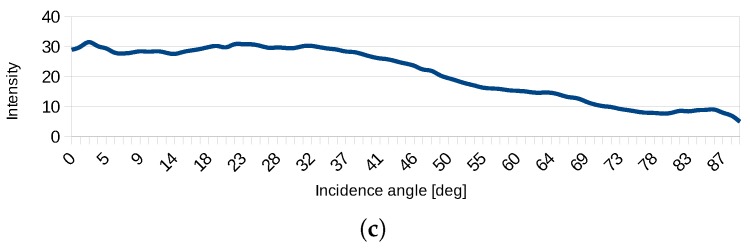
The dependency of laser return intensity on: the source beam (**a**); range of the measurement (**b**); and the angle of incidence (**c**). We are using 2 LiDAR scanners with 16 laser beams per each scanner, 32 beams in total.

Another quality we would like to introduce into the 3D model is the approximate surface “color” information to improve the ability of visual recognition of various objects (inventory, signs, etc.). To avoid additional HW, and preserve invariance to illumination conditions, we use the laser return intensity. However, these intensity values cannot be directly considered as surface reflectivity, since they are affected by various additional factors such as angle of incidence, range of the measurement or gain of the particular laser beam. These factors were reported by previous works [37,38,39] and also confirmed by our experiments in Figure 18.

Previously published works propose various closed-form solutions of intensity normalization for long range measurements (over 10 m) [37,38,39]. However, this is not applicable for smaller indoor environments and therefore we propose an alternative solution. If the normalized intensity represents only the surface reflectivity, there should be no dependency on other factors and probability distribution of the intensities should be the same for different laser beams, angles of incidence, or ranges.

Therefore, we discretize the space of ranges and angles with some small resolution (e.g., 20 cm and 1°>, respectively) and we distribute all the points of the point cloud model into a 3D grid based on the source beam ID (already discrete), the angle of incidence and the range. Our goal is to achieve that the intensity probability distribution will be the same for each bin of points. Assuming normal distribution of surface reflectivities (“colors”), the same target distribution $N(\mu ,\;\sigma^{2})$ will be achieved within each bin by a simple transformation:

(17) $$N\left(\mu ,\;\sigma^{2}\right)=N\left(\mu_{i},\;\sigma_{i}^{2}\right)\cdot \frac{\sigma }{\sigma_{i}}+\left(\mu -\mu_{i}\right),$$

where $N(\mu_{i},\;\sigma_{i}^{2})$ is the original distribution of laser intensities within *i*th bin.

**Figure 19:**
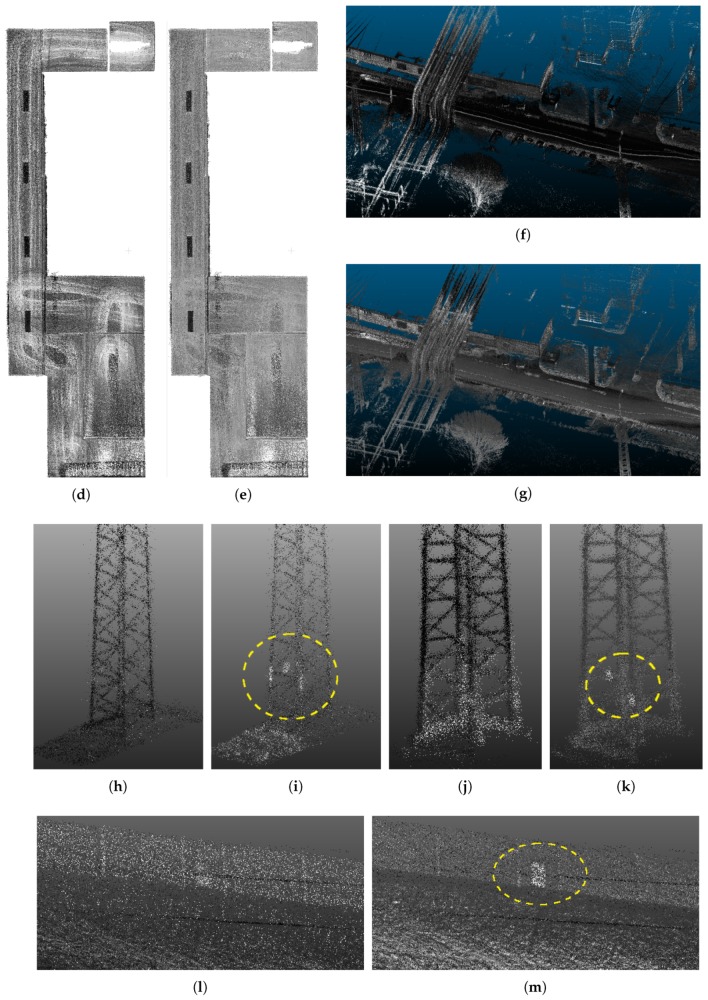
Results of 3D reconstruction without (**a**,**c**,**e**,**g**,**i**) and with (**b**,**d**,**f**,**h**,**j**) the normalization of laser intensities. One can observe more consistent intensities for solid color ceiling (**b**) reducing the artifacts of trajectory, while preserving the contrast with ceiling lights. Besides the consistency, normalization of intensities reduces the noise (**d**). The most significant improvement is the visibility of important objects e.g., markers at the electrical towers (**f**,**h**) or emergency exit doors (**j**) at the highway wall. All these objects can not be found in the original point clouds(**e**,**g**,**i**).

There are no ground truth data to perform any objective evaluation of our proposed method for intensity normalization. We are only able to compare the results of 3D reconstruction with and without the normalization. Examples of results can be found in Figure 19.

## 4. Experiments

This section presents mapping results of our system in various scenes and scenarios—outdoor environments where GNSS is available, indoor scenes with GNSS denied, small rooms, staircases, and a narrow corridor. A usable and precise solution must avoid so called “double walls” described in Figure 3, which are a typical issue in 3D reconstructions causing ambiguity. Unfortunately, evaluation of such duplicities cannot be performed automatically, thus the operator (a certified geodesist) verified the reconstructions for us by inspecting multiple slices across the model. Moreover, the data density and point coloring by the intensity readings are required for better visual recognition of various objects in the environment. All the raw data collected by our backpack solution, and also the 3D reconstructions used in this evaluation, are publicly available (http://www.fit.vutbr.cz/~ivelas/files/4RECON-dataset.zip).

Regarding the precision, our goal is to achieve 5 cm relative precision (e.g., distance of the point from ground truth) denoted as $e_{r}$. For outdoor environments, there are also constraints for absolute error $e_{a}$ in global geodetic frame. The average of this absolute error is required to be below 14 cm for position in horizontal plane and 12 cm for height estimation. However, the constraints for maximal error are set to double of these values—up to 28 cm for horizontal and 24 cm vertical error. These values were obtained through consultation with experts in the field of geodesy and follow the requirements for creating the building models, outdoor vector maps, inventory check, etc. Global error constraints are applicable only outdoors, where some global positioning system is available. To sum up, in this section, we show that our solution provides:

- sufficient relative precision $e_{r}$ under 5 cm;
- global absolute error $e_{a}$ within the limits described above;
- data density and coloring by normalized intensities for visual inspection; and
- data consistency without ambiguity (no dual walls effects).

### 4.1. Comparison of Point Cloud Registration Methods

We compared our previously published CLS method [23] with different modes (online and offline) of state-of-the-art method LOAM [19] using the data of KITTI Odometry Suite [22] providing both the Velodyne LiDAR data and ground truth poses. The error metrics used in this evaluation are defined by the KITTI dataset itself. The data sequences are split into subsequences of 100,200,…,800 frames (of 10,20,…,80 s duration). The error $e_{s}$ of each subsequence is computed as:

(18) $$e_{s}=\frac{{∥E_{s}-C_{s}∥}_{2}}{l_{s}},$$

(provided by [22]) where $E_{s}$ is the expected position (from the ground truth) and $C_{s}$ is the estimated position of the LiDAR where the last frame of subsequence was taken with respect to the initial position (within given subsequence). The difference is divided by the length $l_{s}$ of the followed trajectory. The final error value is the average of errors $e_{s}$ across all the subsequences of all the lengths.

The experiment is summarized in Table 2 and it leads to the conclusion that our CLS approach outperforms LOAM with approximately 1 cm lower drift per 1 m of trajectory elapsed. For clarification, LOAM can run in two different modes. In the online mode (10 fps), mapping is skipped for a certain number of frames, which are only roughly aligned. In the offline mode, which is approximately 3× slower, every frame undergoes the full mapping procedure.

The precision of our method was estimated for frame-to-frame approach, where only consequent frames were registered, and also for the scenario, where each frame is registered with all other frames within a small neighborhood (10 neighboring frames used in this experiment). In this experimental multi-frame approach, the final pose is estimated by simple averaging.

In our previous publication [23], the superior performance of CLS over GICP method (Generalized ICP) [46] was presented, too. All these evaluations led to the choice of CLS for the LiDAR frames registration in our 4RECON backpack solution.

### 4.2. Indoor Experiments

**Figure 20:**
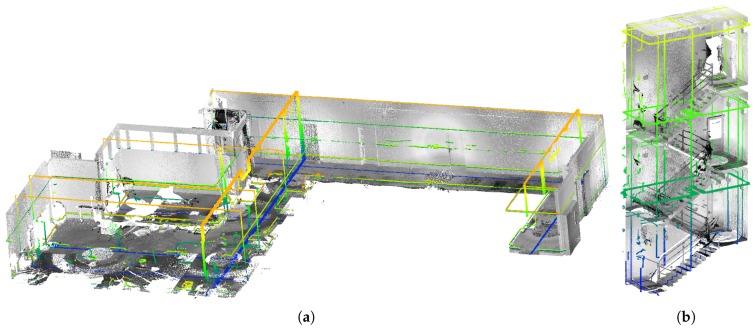
Experimental environments Office (**a**) and Staircase (**b**), and the highlighted slices that were used for precision evaluation.

For indoor evaluation of our system, we chose two different environments—the office and staircase in Figure 20—where our partner company has already performed 3D mapping using different laser scanners and generously provided the accurate output models to us. The reconstructions from static *FARO* scanner achieving very high accuracy (in order of millimeters) were used as the ground truth. The same strategy has been already used for evaluation of other mapping systems [4,12,25]. For the office environment only, also the 3D reconstruction created by *ZEB-1* solution was provided to us. This allowed us to compare our solution in terms of accuracy, data density, model usability and completeness.

**Figure 21:**
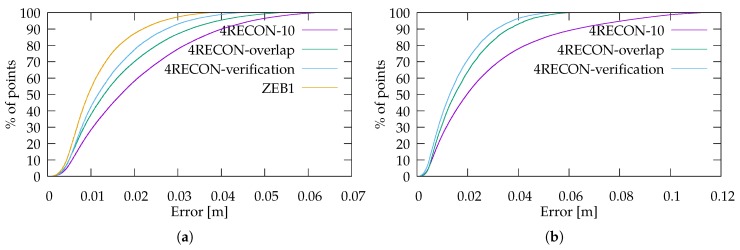
Error $e_{r}$ distribution (the amount of the points within certain error) for our system 4RECON and ZEB-1 product. The experiments were performed for all test slices in Figure 20 on Office (**a**) and Staircase (**b**) dataset. Note that the model built by ZEB-1 was not available and therefore the evaluation is missing.

To evaluate the relative error, all the models of the same environment provided by different scanners (FARO, ZEB-1, and our solution 4RECON) were aligned using ICP. As displayed in Figure 20, several reference slices (8 slices per model, 16 slices in total) were created for the evaluation of precision. Within each slice, the average error (in Table 3) was estimated as the average distance of the 3D points to the ground truth model created by the FARO scanner. Our solution achieved approximately 1.5 cm relative error on average, which is only slightly worse result than 1.1 cm error for ZEB-1 that is burdened by the multiple limitations listed below in this section. Moreover, we provide information about the distribution of displacement relative error in Figure 21. The error was estimated for ZEB-1 and different modes of our system:

- in *4RECON-10*, the registrations were performed only within small neighborhood of 10 nearest frames (1 s time window) and reflects the impact of accumulation error;
- for *4RECON-overlap*, the registrations were performed for all overlapping frames as described in Section 3.7 reducing the accumulation error by loop closures at every possible location; and
- pose graph verification (see Section 3.8) was deployed in *4RECON-verification*, yielding the best results with good precision and no ambiguities.

Both ZEB-1 and our solution including pose graph verification achieved sufficient accuracy below 5 cm. Moreover, the precision of 2 cm was fulfilled for more than 70% of data. Slightly better precision of ZEB-1 solution was achieved thanks to the Hokuyo sensor with 4× higher scanning frequency while preserving much lower vibrations compared with Velodyne LiDAR.

**Figure 22:**
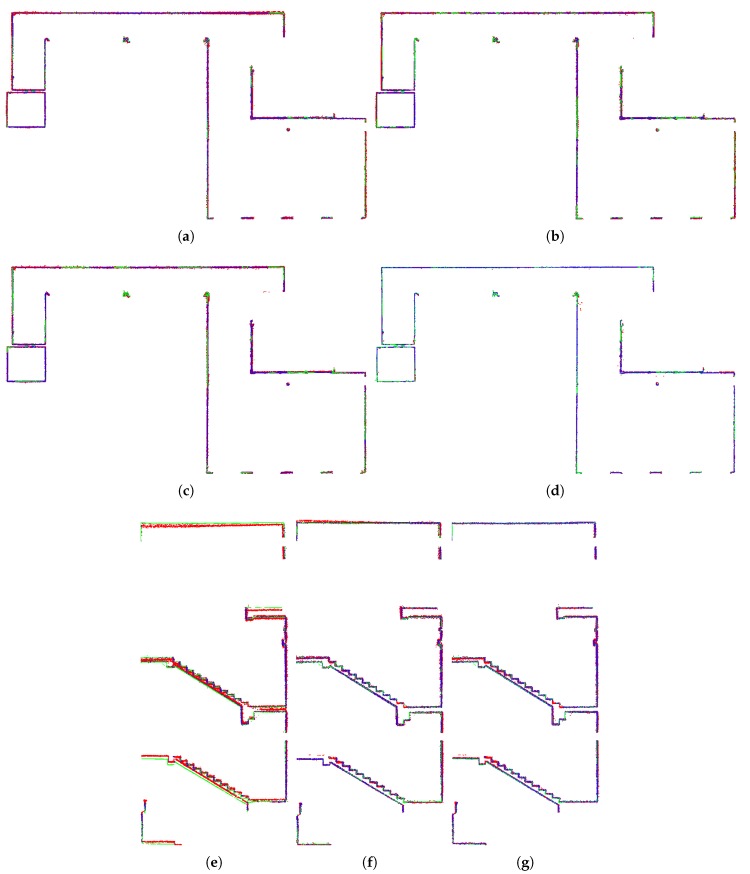
Color coded errors within the horizontal reference slice of the Office dataset (**a**)–(**d**) and vertical slice in Staircase dataset (**e**)–(**g**). Blue color represents zero error, red color stands for 10 cm error and higher. The ground truth FARO data are displayed in green. The results are provided for 4RECON-10 (**a**,**e**), 4RECON-overlap (**b**,**f**), 4RECON-verification (**c**,**g**), and ZEB-1 (**d**). For Office dataset, there are no ambiguities (double walls) even without visual loop detection while both loop closure and pose graph verification is necessary for more challenging Staircase dataset to discard such errors. Moreover, one can observe that ZEB-1 solution yields lower noise reconstruction thanks to the less noisy Hokuyo LiDAR.

Figure 22 also shows the precision within representative slices—horizontal slice for Office dataset and vertical slice across model of Staircase. These slices demonstrate the noise within data coming from different sensors—Hokuyo LiDAR for ZEB-1 solution and Velodyne for our 4RECON system—and also the precision for different modes of operation. For Staircase dataset, the necessity of pose graph optimization is also demonstrated.

Our evaluations show that the precision of our 4RECON backpack is comparable to the solution ZEB-1 while fulfilling basic requirement for relative error below 5 cm. Note that the error values are also comparable (and in some cases better) to the precisions of other solutions in Table 1. In our solution, higher noise can be observed comparing with ZEB-1. This corresponds with higher error values and it is the main reason for little lower accuracies.

However, it is important to point out two most significant advantages of our solution comparing with ZEB solutions. First, our solution is *usable in vast open spaces* with fewer and more distant featuring objects, as is demonstrated in the next sections. In indoor environments featuring objects at distances significantly larger than 15–20 m [7], ZEB solutions based on the Hokuyo sensor fail.

**Figure 23:**
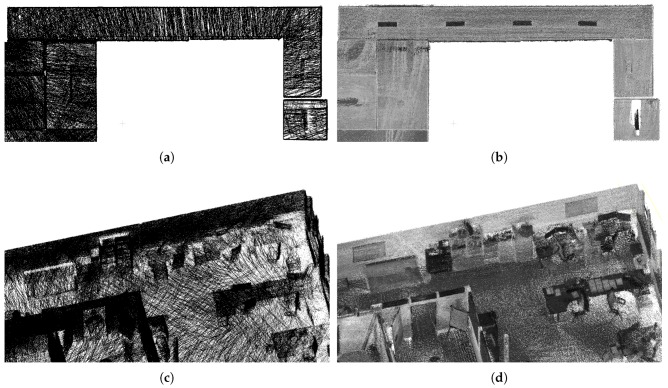
The comparison of data density provided by ZEB-1 (**a**,**c**) and our (**b**,**d**) solution. Since the ZEB-1 solution is based on the Hokuyo scanner, the laser intensity readings are missing and data density is much lower compared with our solution. Multiple objects which can be distinguished in our reconstruction (lamps on the ceiling in the top, furniture and other equipment in the bottom image) are not visible in the ZEB-1 model.

Second, our Velodyne-based solution is able to provide much higher *data density, map completeness and visibility of objects* in the scene. We chose two large surfaces (the ceiling and the side wall in Figure 23) with 230 m^2^ in total area. Models of these surfaces created by ZEB-1 solution achieved average data density 0.9 points per cm^2^ (2.2 million points in total). Models created by our 4RECON backpack consist of more than 23 million points, achieving much higher data density—10.1 points per cm^2^. Better visibility of objects in Figure 23 is achieved thanks to the laser intensity readings provided by Velodyne sensor and employing our normalization process as described in the Section 3.10. This might appear to be only a “cosmetic” property, but the visibility of the construction elements, equipment, furniture, etc. in the scene is important for usability in real applications—e.g., an operator needs to distinguish between the window and the blackboard.

### 4.3. Outdoor Experiments

**Figure 24:**
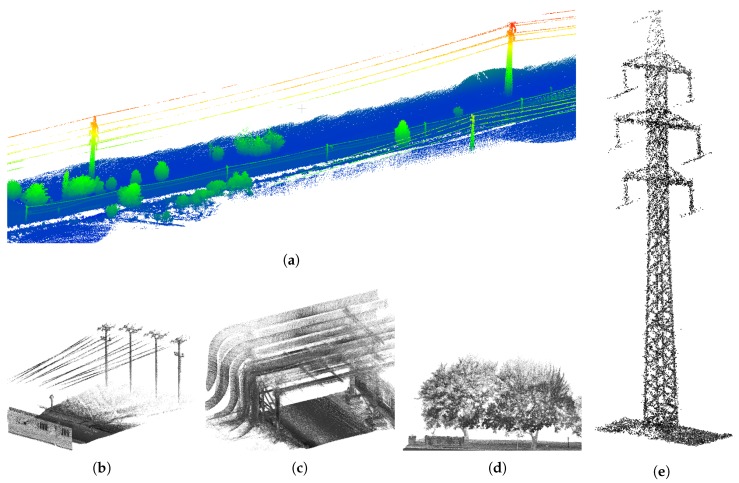
The example of 3D reconstruction of open field with high voltage electrical lines (**a**). The model is height-colored for better visibility. The estimation of positions and height of the lines (**b**), towers (**e**), etc. was the main goal of this mapping task. The other elements (**c**,**d**) in the scene are shown for demonstration of the reconstruction quality.

Our system is a universal solution—both for indoor scenes, where the usability was proven by the previous section, and for outdoor scenes, including vast open ones. We tested and evaluated our system during a real task—high voltage lines mapping and measurement. The area of interest, including the details of some important objects, is visualized in Figure 24. The main goal of this mission was position estimation of electric pylons (including footprint of the base, total height and the positions of the wire grips) and the heights and the hangings of the wires. Figure 24 shows that these details can be recognized in the 3D model. The usability of our 3D reconstructions was also confirmed by the geodetic company we asked for manual data inspection and evaluation.

**Figure 25:**
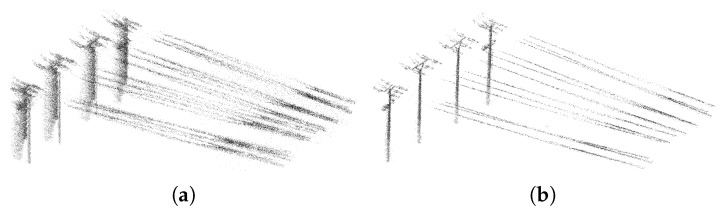
Example of ambiguities caused by reconstruction errors (**a**), which disqualifies the model to be used for practical measurements. We obtained such results when we used only poses provided by GNSS/INS subsystem without any refinements by SLAM or point cloud registration. Our solution (including SLAM) provides valid reconstructions (**b**), where both towers and wires (in this case) can be distinguished.

In the same way as during the indoor mapping, the ambiguities in multiple instances of objects disqualifies the reconstructions to be used in practical geodetic measurements. Such error in comparison with the desired result of the reconstruction is shown in Figure 25. Multiple instances of the same object, blurred and noisy results were successfully avoided by our solution (see Figure 24 and Figure 25).

Since our solution integrates precise GNSS/INS module for outdoor scenarios, the model is *georeferenced*—the coordinates of all the points are bound in some global geodetic frame.

To verify the absolute positional accuracy of our model, we performed precise measurements on so-called survey markers. This is commonly used technique to verify the precision of resulting maps (including 3D maps). Precise positions of the survey markers are estimated using specialized geodetic GNSS system, which is placed statically on the survey point for several seconds, until the position converged. The precision up to 2 cm is achieved using RTK (Real Time Kinematics) which are received online via internet connection.

**Figure 26:**
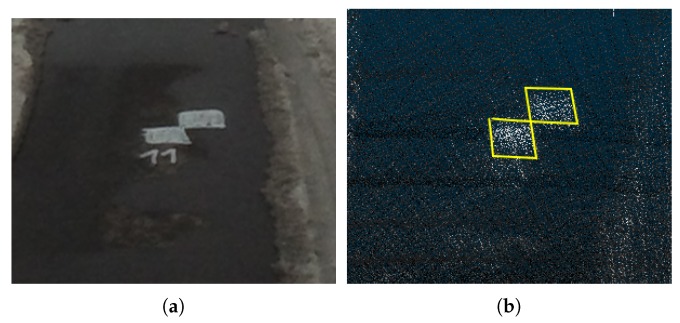
Geodetic survey markers painted on the road (**a**) is also visible in the point cloud (**b**) thanks to the coloring by laser intensities.

Survey markers (Figure 26a) are highlighted using high-reflective sprays. Thanks to the coloring of point cloud by laser intensities, these markers are also visible in the reconstructions as can be seen in Figure 26b.

The evaluation in Table 4 shows that our 3D mapping for 0.5 km test track fulfills the requirements for absolute error, as described at the beginning of this section—average error below 14 cm for position in horizontal plane and 12 cm for height estimation and maximal error up to 28 cm and 24 cm, respectively (double values of expected average error).

Thanks to the ability of point cloud coloring by laser intensities, it is possible to also run such evaluation for the validation of each 3D model, which should be used in real application. This is also an important quality, since there are requirements for double measurements in geodesy to ensure that the accuracy is sufficient.

### 4.4. Comparison of Single and Dual Velodyne Solution

**Figure 27:**
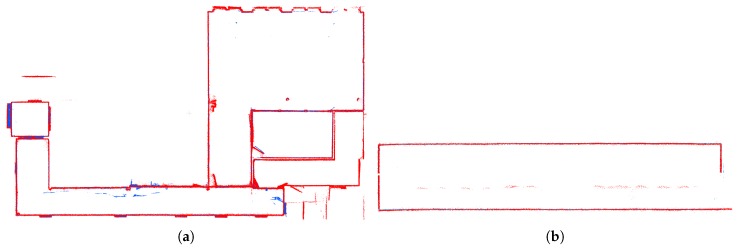

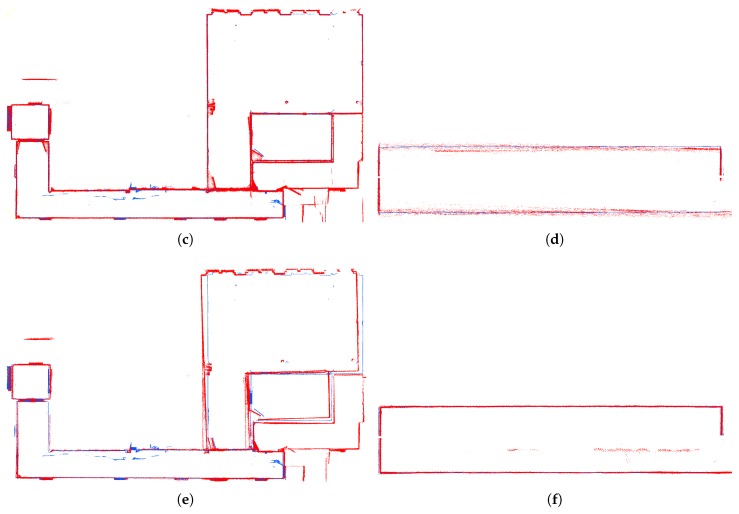
Comparison of reconstructions provided by dual LiDAR system—floor plan top view (**a**) and side view of the corridor (**b**)—with the reconstruction built using only single horizontally (**c**,**d**) or vertically (**e**,**f**) positioned Velodyne LiDAR. The reconstructions are red colored with ground truth displayed in blue.

Finally, we compared the robustness of our dual LiDAR solution over the system with single LiDAR only. We computed reconstructions of the Office environment using our solution with two synchronized and calibrated LiDARs (one aligned vertically and second horizontally) in Figure 27a,b and also using only single LiDAR—horizontally ( Figure 27c,d) or vertically aligned ( Figure 27e,f).

Our evaluation shows that the dual LiDAR solution provides a valid reconstruction. However, the solution with horizontal LiDAR only is not able to provide vertically correct alignment (Figure 27d), and vice versa, the solution with vertical LiDAR is horizontally misaligned (Figure 27e).

## 5. Discussion

When we look on our 4RECON mapping backpack in the context of the other available solutions (see overview in Table 1), we can summarize its advantages and disadvantages.

Comparing to the ZEB products, our backpack achieves much higher data density, better visibility of the objects in the resulting model, higher comfort of data acquisition, and, most importantly, usability also in the outdoor featureless open spaces, including the option of georeferencing the reconstructed point map. However, we must admit that ZEB scanners achieve better accuracy and lower noise in the models of indoor environments.

In terms of universality of the usage, our solution also outperforms Robin and Akhka backpacks, which require GNSS readings and therefore indoor scanning is not possible. For outdoor tasks, Robin achieves better precision than our 4RECON backpack, but it is also important to point out the very high price of the Robin solution.

Laser mapping backpacks Pegasus, Viametris bMS3D and LiBackpack can be considered as the most similar solutions to our work. All these systems claim precision up to 5 cm, which is also the accuracy of 4RECON (according to the evaluation in Figure 20). The advantages of these solutions are more professional design and the presence of additional RGB cameras (for Pegasus and Viametris backpacks). The integration of panoramic RGB camera into our backpack is the plan for future work. Our solution on the other side provides open SLAM method in comparison with the proprietary solutions deployed in these backpacks, and also potentially much lower price.

## 6. Conclusions

This paper presents a dual LiDAR system for mobile mapping. Our solution can be easily carried as a backpack together with a reliable dual antenna GNSS/INS system. This leads to the universality of its usage. In small or narrow indoor environments with many obstacles, two LiDAR sensors increase the field of view. On the other side, in open outdoor spaces with lack of features, the reliable positional subsystem keeps the result accurate.

Thanks to the type of LiDARs used, our solution also brings multiple other beneficial properties: data density, map completeness and coloring by laser intensities normalized by our novel algorithm. The intensities enables better visual recognition of the elements in the scene as well as the visibility of geodetic survey markers for checking the model validation.

The proposed solution was evaluated in both indoor and outdoor scenarios. During the mapping of the office or staircase environment, our solution fulfilled the requirement of error below 5 cm and achieved a similar precision as solution ZEB-1. The average error in terms of the points displacements is approximately 1.5 cm. For outdoor experiments, our reconstruction met the requirements for absolute precision with 11.8 cm average error in the global geodetic frame. This proves higher universality of our mapping backpack compared to the previous ZEB-1 solution. In all our experiments, data consistency was preserved and unambiguous models were built.

## Figures and Tables

**Table 1:** Overview of related LiDAR mobile mapping solutions.

Solution (Released in)		Sensor (Precision)	Range	System Precision	Price €	Open Method	Properties and Limitations	Intensities
ZEB-1 (2013)	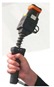 [3]	Hokuyo UTM-30LX (3 cm up to 10 m range)	15–20 m (max 30 m under optimal conditions)	up to 3.8 cm indoors [4]	N/A	Proprietary, based on [5,6]	missing (laser) intensity readings no GNSS reference requires visible featuring objects at close distances	No
ZEB-REVO (2015) [7]	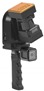 [3]	Hokuyo UTM-30LX-F (3 cm up to 10 m range)	15–20 m (max 30 m under optimal conditions) [7]	up to 3.6 cm indoors [8]	34,000	Proprietary, based on [5,6]	missing (laser) intensity readings no GNSS reference requires visible featuring objects at close distances	No
LiBackpack (2019) [9]	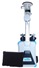 [10]	2× Velodyne VLP-16 (3 cm)	100 m (Velodyne scanner limitation)	5 cm	60,000	Proprietary	intensity readings available GNSS support dual LiDAR (one for odometry only, second for reconstruction)	Yes
Pegasus (2015) [11]	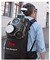 [11]	2× Velodyne VLP-16 (3 cm)	50 m usable range	5 cm with GNSS (5–50 cm without). 4.2 cm in underground bastion [12]	150,000	Proprietary	intensity readings available GNSS support dual LiDAR (cooperation unknown)	Yes
Viametris bMS3D [13,14]	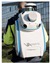 [14]	2× Velodyne VLP-16 (3 cm)	100 m (Velodyne scanner limitation)	5 cm under appropriate satellite reception conditions	N/A	Proprietary	intensity readings and RGB coloring available GNSS support dual LiDAR (cooperation unknown)	Yes
Robin (2016) [15]	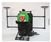 [16]	RIEGL VUX-1HA (3 mm)	120/420 m in slow/high frequency mode (for sensor)	up to 3.6 cm at 30 m range (FOG IMU update)	220,000	Proprietary	intensity readings dual GNSS (at least weak) GNSS signal required	Yes
Akhka (2015) [17,18]	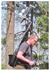 [17]	FARO Focus3D 120S (1 mm)	120 m (sensor range)	8.7 cm in forest environments	N/A	Open [17]	intensity readings outdoor only (GNSS required)	Yes

**Table 2:** Comparison of visual odometry error for SoA method LOAM and our CLS method. The experiments were performed on KITTI Odometry dataset [22]. For CLS, frame to frame (single) or frame to multiple (10) neighboring frames (multi-frame) registrations without any loop closures were performed. In LOAM experiments, both the original online version (providing real time performance) and offline version (with full procedure for each frame omitting approximations) was used. In all data sequences, except the short sequence No. 4 where the car drives only forward without any turns, our multi frame approach outperformed the LOAM solution.

		Error *e_s_* (18)			
Sequence	Length	LOAM Online	LOAM Offline	CLS Single	CLS Multi-Frame
0	4540	0.052	0.022	0.022	**0.018**
1	1100	0.038	0.040	0.042	**0.029**
2	4660	0.055	0.046	0.024	**0.022**
3	800	0.029	0.019	0.018	**0.015**
4	270	0.015	**0.015**	0.017	0.017
5	2760	0.025	0.018	0.017	**0.012**
6	1100	0.033	0.016	0.009	**0.008**
7	1100	0.038	0.019	0.011	**0.007**
8	4070	0.035	0.024	0.020	**0.015**
9	1590	0.043	0.032	0.020	**0.018**
**Weighted average**	2108	0.043	0.029	0.022	**0.017**

**Table 3:** Relative error $e_{r}$ of our method and ZEB-1 product within selected slices visualized in Figure 20. Presented values are average displacements (cm) of the points comparing with the ground truth point cloud obtained by FARO static scanner. The results are missing for ZEB-1 and Staircase dataset since there was no reconstruction using this scanner available.

Dataset	Slice #	4RECON-10	4RECON-Overlap	4RECON-Verification	ZEB-1
**Office**	1	2.50	1.71	1.49	1.44
	2	1.97	1.47	1.31	1.06
	3	1.70	1.75	1.55	1.22
	4	1.82	1.54	1.31	1.22
	5	1.93	1.63	1.53	1.44
	6	2.13	1.49	1.47	1.29
	7	2.09	1.68	1.37	0.97
	8	2.07	1.36	1.37	1.31
	Average $e_{r}$ (cm)	2.01	1.62	1.41	1.14
**Staircase**	1	3.23	2.11	1.81	-
	2	3.99	1.87	1.60	-
	3	2.63	1.65	1.61	-
	4	2.74	1.71	1.53	-
	5	2.42	1.68	1.50	-
	6	2.98	2.67	1.67	-
	7	1.76	1.75	1.29	-
	8	1.82	1.67	1.56	-
	Average $e_{r}$ (cm)	2.74	1.82	1.57	-

**Table 4:** Errors measured (cm) on geodetic survey marker points at the beginning and at the end of survey track. The distance between the control points is 523 m.

Ref. Point	dX	dY	Horizontal Error	dZ (Vertical)	Total Error $e_{a}$
1	−5.9	−1.2	6.0	−15.2	16.3
2	−5.6	0.5	5.6	−4.7	7.3
